# Association between Mental Health Knowledge Level and Depressive Symptoms among Chinese College Students

**DOI:** 10.3390/ijerph18041850

**Published:** 2021-02-14

**Authors:** Shuo Cheng, Di An, Zhiying Yao, Jenny Jing-Wen Liu, Xuan Ning, Josephine Pui-Hing Wong, Kenneth Po-Lun Fung, Mandana Vahabi, Maurice Kwong-Lai Poon, Janet Yamada, Shengli Cheng, Jianguo Gao, Xiaofeng Cong, Guoxiao Sun, Alan Tai-Wai Li, Xinting Wang, Cunxian Jia

**Affiliations:** 1 School of Public Health, Cheeloo College of Medicine, Shandong University, Jinan 250012, China; chengshuo316@163.com (S.C.); asandi.1993@163.com (D.A.); yaozhiyingsdu@163.com (Z.Y.); wxt@sdu.edu.cn (X.W.); 2 Daphne Cockwell School of Nursing, Ryerson University, Toronto, ON M5B 3K2, Canada; jenny.liu@psych.ryerson.ca (J.J.-W.L.); xuan.ning@ryerson.ca (X.N.); jph.wong@ryerson.ca (J.P.-H.W.); mvahabi@ryerson.ca (M.V.); janet.yamada@ryerson.ca (J.Y.); 3 Department of Psychiatry, University of Toronto, Toronto, ON M5S 1A1, Canada; ken.fung@uhn.on.ca; 4 Asian Mental Health Initiative, Toronto Western Hospital, Toronto, ON M5T 2S8, Canada; 5 School of Social Work, York University, Toronto, ON M3J 1P3, Canada; mklpoon@yorku.ca; 6 School of Philosophy and Social Development, Shandong University, Jinan 250100, China; chengsl@sdu.edu.cn (S.C.); gaoj@sdu.edu.cn (J.G.); 7 School of Political Science and Law, Jinan University, Jinan 250022, China; jndxcxf@sina.com; 8 School of Physical Education, Shandong University, Jinan 250061, China; sunguoxiao@sdu.edu.cn; 9 Primary Care, Regent Park Community Health Centre, Toronto, ON M5A 2B2, Canada; alanli@inspiract.com

**Keywords:** college students, mental health, knowledge, depressive symptoms, China

## Abstract

This study aimed to explore the association between mental health knowledge level and the prevalence of depressive symptoms among Chinese college students. A cross-sectional study was conducted in six universities in Jinan, Shandong Province, China, and a total of 600 college students were recruited to self-complete a series of questionnaires. The Mental Health Knowledge Questionnaire (MHKQ) was used to investigate the level of mental health knowledge. Depressive symptoms were investigated with the depression subscale of the Depression Anxiety Stress Scale (DASS-21). The prevalence rate of depressive symptoms among college students was 31.2%. Compared with MHKQ scoring in the 1st quartile, college students with MHKQ scoring in the 3rd quartile and in the 4th quartile reported lower levels of depressive symptoms after adjusting for potential confounding factors. Since mental health knowledge level was related to depressive symptoms among college students, increased efforts to promote the level of mental health knowledge in Chinese college students are critical.

## 1. Introduction

College students are a key population closely related to the progress and development of the future society. Faced with changing environments, college students experience increased stressors, thereby increasing the risks of experiencing depressive symptoms and other mental health problems [1,2]. Relevant research has indicated a high prevalence of depressive symptoms among college students [3,4,5]. A meta-analysis of the occurrence of depressive symptoms among Chinese college students has revealed that the prevalence rate of depressive symptoms was around 24% [6]. A study investigating college students in 26 countries in Africa, Asia, the Caribbean, and Latin America has reported similar prevalence rates, with 24.0% noted as moderate and 12.8% as severe [7]. Depressive symptoms have a negative impact on academic achievement [8], interpersonal relationships, and the daily life [9] of college students. Furthermore, depressive symptoms have been reported to increase the risk of suicidal ideation or behavior [10,11,12]. As a result, depressive symptoms are a major problem among college students [13,14], and it is necessary to explore the factors related to depressive symptoms.

Mental health literacy, proposed by Jorm, refers to the knowledge and beliefs that help individuals to identify, prevent, and cope with mental disorders [15]. China has addressed the issue of mental health literacy in national health plans since 2002 [16]. The most recent mental health plan, covering 2015–2020, specified that there should be a general improvement in the public’s mental health knowledge [17]. Mental health knowledge includes the following components: general mental health knowledge, knowledge on the prevention of mental health problems, knowledge on the causes of mental health problems, and mental health promotion activities [18]. Relevant research has revealed that the awareness rate of mental health knowledge among Chinese college students needs to be further strengthened [19].

Although the prevalence rates of mental health problems among college students are high, the proportion of students who are aware of early mental health symptoms and seek help from professionals or institutions is low [20,21]. Researchers state that the lack of mental health knowledge is one of the most important reasons for this problem [22,23]. Improved mental health knowledge may be able to promote early identification of mental health problems and increase the use of health services [24]. At present, there are few studies on the association between mental health knowledge level and depressive symptoms among college students. We hypothesized that lower depressive symptoms were associated with higher mental health knowledge level. The specific objectives of the present study were as follows: (1) to investigate the level of mental health knowledge and depressive symptoms among college students; and (2) to analyze the association between mental health knowledge level and depressive symptoms among college students.

## 2. Materials and Methods

### 2.1. Participants and Data Collection Procedure

A cross-sectional study was conducted from 25 April to 4 June 2019 in six universities in Jinan, Shandong Province, China. We respectively selected two universities each from the central area, the eastern part, and the western part of Jinan. We recruited 600 college students (100 per university) to take part in this survey. Inclusion criteria for participation were: full-time enrollment as a student, 18 years of age or older, freshmen to seniors, and attending one of the six partnering universities in Jinan, Shandong Province, China.

Participating students completed the questionnaires via an online platform Questionnaire Star. Informed consent was obtained prior to the start of the survey. Investigators were trained to understand the purpose of the study and the content of the questionnaires. The survey package was distributed via an online link in class, where investigators also explained the anonymity and confidentiality of the survey to participating students. Participants were uniformly instructed to fill in the questionnaire. Data were then imported from Questionnaire Star onto SPSS for cleaning and analysis.

### 2.2. Measures

#### 2.2.1. General Demographic Characteristics

A self-designed general information questionnaire was used to investigate demographic characteristics. Demographic information included age, gender, only child or not, family origins, physical health status, sleep quality, academic performance, parents’ physical health status, parents’ mental health status, parents’ marital status, etc. Specifically, physical health status was measured by “How would you rate your physical health?” (Good/Fair/Poor). Sleep quality was assessed by one question: “How would you rate your satisfaction with your sleep?” (Good/Fair/Poor). Academic performance was asked by “Compared to other students, how would you rate your overall academic performance?” (Good/Fair/Poor). Parents’ physical health status was measured by “How would you rate your father’s overall physical health?” and “How would you rate your mother’s overall physical health?” (Good/Fair/Poor/Unknown). Parents’ mental health status was assessed by “How would you rate your father’s overall mental health?” and “How would you rate your mother’s overall mental health?” (Good/Fair/Poor/Unknown). Parents’ marital status was classified as “Harmonious”, “Sometimes quarrel”, and “Others” (including separated, divorced, and one or both parents passed).

#### 2.2.2. The Assessment of Mental Health Knowledge Level

The 20-item Mental Health Knowledge Questionnaire (MHKQ) was used to evaluate the mental health knowledge level of college students. The MHKQ was developed by the National Health Commission of China, and has been widely used to assess mental health knowledge level among Chinese adults [25,26]. Items 1–16 were answered by “Yes” or “No”, and each item was scored 1 for correct answer, and 0 for wrong answer. Items 17–20 were answered by “Know” or “Don’t know”, and each item was scored 1 for “Know”, and 0 for “Don’t know”. Overall awareness rate of mental health knowledge was calculated as follows: sum of items correctly answered by all individuals/(total number of participants × total number of items) × 100%. The overall mental health knowledge score was calculated by summing the score of each item. A high score on the MHKQ indicated greater mental health knowledge level. In this study, the Cronbach’s α coefficient of MHKQ was 0.6.

#### 2.2.3. The Assessment of Depressive Symptoms

Depressive symptoms were measured with the depression subscale of the 21-item Depression Anxiety Stress Scales (DASS-21) containing seven items. The DASS-21 has been widely used among Chinese college students [27,28,29]. According to the feelings of the participants in the last week, each item within the subscale was scored on a four-point Likert-type scale (0 = never, 1 = sometimes, 2 = often, 3 = always). A score on the depression subscale ≥10 indicated the presence of depressive symptoms. Scores of all items in the scale were multiplied by 2 to give the final score. The severity of depressive symptoms was categorized into five scales: normal (0~9), mild depressive symptoms (10~13), moderate depressive symptoms (14~20), severe depressive symptoms (21~27), and extremely severe depressive symptoms (28~42) [30]. In this study, the Cronbach’s α coefficient of DASS-21 was 0.8.

### 2.3. Statistical Analysis

The Statistical Package for Social Sciences (SPSS) 24.0 software (IBM Corp, Armonk, New York, NY, USA) was used for statistical analysis. Continuous variables were tested for normality. Continuous variables were described in mean (Standard Deviation, *SD*) when conforming to normal distribution, and described by median and quartile (*Q*_1_, *Q*_3_) when not conforming to normal distribution. Classification variables were denoted by *n* (%). Chi-square analysis was used to examine the frequency and distribution of depressive symptoms in various demographic groups. The association between mental health knowledge level and depressive symptoms among college students was analyzed by unconditional logistic regression. Values of *p* < 0.05 were considered statistically significant.

## 3. Results

### 3.1. Demographic Characteristics of the Participants

In this study, 600 participants with a mean age of 19.5 ± 1.3 were surveyed. As shown in Table 1, there were 265 (44.2%) only children and 335 (55.8%) non-only children. The ratio of males to females was 1 to 1. There were 242 (40.3%) students from the city and 358 (59.7%) students from rural areas. Over half of the participants surveyed reported to be in good health (58.6%) and slept well (52.0%). Over half evaluated their academic performance as fair (58.8%). The majority of the students considered their fathers to be in a good physical health status (53.3%), and good mental health status (69.7%). Students who thought their mothers were in a good physical health status accounted for 46.7%, and in good mental health status accounted for 67.2%. Most of the students surveyed reported their parents’ marital status as good (Harmonious: 55.8%, Sometimes quarrel: 37.5%).

### 3.2. Mental Health Knowledge Level among College Students

The scores of the MHKQ ranged from 9.0 to 20.0, with a median score of 17.0. Quartile 1 was 15.0, quartile 3 was 18.0, and the interquartile range (*Q*_3_–*Q*_1_) was 3.0. The awareness rate of mental health knowledge among college students was 83.0%. Across genders, rates significantly differed, with males’ mental health knowledge rates at 81.2%, and females’ at 84.8% (*χ*^2^ = 26.62, *p* < 0.001).

### 3.3. Depressive Symptoms among College Students

The scores of the depression subscale of DASS-21 ranged from 0 to 38.0, the median of score was 4.0, and the interquartile range (*Q*_3_–*Q*_1_) was 8.0. There were 187 college students whose scores reached the threshold for depressive symptoms, accounting for 31.2% of the total students. Among those, 83 students reported mild depressive symptoms (13.8%), 73 students reported moderate depressive symptoms (12.2%), 23 students reported severe depressive symptoms (3.8%), and 8 students reported extremely severe depressive symptoms (1.3%). There were 93 male students and 93 female students with depressive symptoms, accounting for 31.1% among both male students and female students.

### 3.4. Associated Factors of Depressive Symptoms among College Students

Chi-square analysis showed that depressive symptoms among college students were statistically significant with family origins (χ^2^ = 8.87, *p* = 0.003), physical health status (χ^2^ = 48.83, *p* < 0.001), academic performance (χ^2^ = 38.80, *p* < 0.001), sleep quality (χ^2^ = 33.51, *p* < 0.001), father’s physical health status (χ^2^ = 14.92, *p* = 0.001), father’s mental health status (χ^2^ = 18.40, *p* < 0.001), mother’s physical health (χ^2^ = 28.41, *p* < 0.001), mother’s mental health status (χ^2^ = 27.56, *p* < 0.001), parents’ marital status (χ^2^ = 12.80, *p* = 0.002), and not statistically significant with only child or not (χ^2^ = 0.92, *p* = 0.337) and gender (χ^2^ = 2.48, *p* = 0.629). Detailed results are represented in Table 2.

### 3.5. Association between Mental Health Knowledge Level and Depressive Symptoms among College Students

Logistic regression analysis showed that college students with MHKQ scoring in the 3rd quartile (17~18) (OR = 0.56, 95%CI = 0.31~0.98) and the 4th quartile (18~20) (OR = 0.60, 95%CI = 0.36~0.99) were associated with a lowered risk of depressive symptoms, compared with MHKQ scoring in the 1st quartile (0~15). After adjusting for personal associated factors such as family origins, physical status, sleep quality, and academic performance, college students with MHKQ scoring in the 3rd quartile (17~18) (OR = 0.52, 95%CI = 0.28~0.98) and the 4th quartile (18~20) (OR = 0.52, 95%CI = 0.30~0.91) were associated with a lowered risk of depressive symptoms, compared with MHKQ scoring in the 1st quartile (0~15). After adjusting personal associated factors and family-associated factors such as parents’ physical status, parents’ mental health status, and parents’ marital status, college students with MHKQ scoring in the 3rd quartile (17~18) (OR = 0.52, 95%CI = 0.27~0.99) and the 4th quartile (18~20) (OR = 0.53, 95%CI = 0.30~0.97) were associated with a lowered risk of depressive symptoms, compared with MHKQ scoring in the 1st quartile (0~15). More details are shown in Table 3.

## 4. Discussion

In this study, the major findings are as follows: (1) The prevalence rate of depressive symptoms was high among Chinese college students. There was no gender-based difference in depressive symptoms between male students and female students. (2) The awareness rate of mental health knowledge was 83.0%. The mental health knowledge level of female students was higher than that of male students. (3) Mental health knowledge level was associated with depressive symptoms among Chinese college students, and college students with higher mental health knowledge level reported lower levels of depressive symptoms.

College students are prone to depressive symptoms and other psychological problems after entering post-secondary studies [31]. This study showed that college students with depressive symptoms accounted for 31.2%, which was similar to the results found with Australian college students [32], but higher than those reported from a meta-analysis of the prevalence of depressive symptoms among Chinese college students (23.8%) [6], and higher than the prevalence rates of depressive symptoms among college students in Hunan, China (18.6%) [33]. There were no statistically significant differences in the ratio of depressive symptoms between male and female college students, which is consistent with previous research [34,35]. Our study results suggest that college students’ vulnerability to psychological problems should not be ignored and needs to be recognized by individuals, schools, families, and society.

The current study found that the awareness rate of mental health knowledge among college students was 83.0%. The awareness rate of mental health knowledge among college students in this study was higher than the result of a systematic review of mental health knowledge among Chinese college students (73.0%) [19]. The awareness rate of mental health knowledge among female students was higher than that among male students, which is consistent with previous research [36]. Findings can be attributed to the increased awareness and monitoring of signs and symptoms of mental health problems observed in females, whereas males were noted to be less aware of mental health problems [37]. However, there were also studies demonstrating no differences with respect to gender [26]. Thus, gender differences may be an issue to be further explored in the context of developing effective mental health education and promotion programs.

This was the first study that examined the association between mental health knowledge level and depressive symptoms among Chinese college students. To date, there has only been one relevant study conducted among left-behind middle school students [38], which found an association between higher awareness of mental health knowledge and better mental health overall. The current study found consistent results among college students, that is, depressive symptoms were negatively associated to mental health knowledge level among college students, and college students with higher mental health knowledge reported lower levels of depressive symptoms. This may be due to mental health knowledge being related to one’s attitude and coping with mental health problems.

Studies have showed that increases in mental health knowledge may be an effective strategy to reduce the stigma of mental health and improve help-seeking behaviors [39,40]. College students with higher mental health knowledge may better understand the contributing factors or better recognize the triggers of psychological problems [19]. This study has established that the levels of mental health knowledge were associated with depressive symptoms among Chinese college students. Thus, education programs that increase mental health knowledge are critical in improving mental health among college students [24].

There are some limitations to this study. First, the research participants were selected from six universities in Jinan; generalization of the results to college students in other regions may be limited and a larger range of sample surveys could be carried out in the future. Second, in this study, we used a cross-sectional design. Although we explored the association between mental health knowledge level and depressive symptoms, we could not sufficiently verify the causal association. Longitudinal studies should be carried out in the future to further examine the causal or long-term associations between mental health knowledge and depressive symptoms among college students. In addition, depressive symptoms and mental health knowledge were assessed by self-reported questionnaires, and information bias may exist related to participants’ recollection of information or their unwillingness to disclose information.

## 5. Conclusions

Depressive symptoms are commonly reported among college students. Findings from the current study provide a better understanding of the associated factors of depressive symptoms and broaden our understanding of their views. To our knowledge, this study is the first to establish an association between levels of mental health knowledge and depressive symptoms among Chinese college students. College students with higher mental health knowledge level reported lower levels of depressive symptoms. These findings serve as an important reference for developing policy and practice to promote mental health among college students. Mental health education should pay more attention to improving mental health knowledge level among college students.

## Figures and Tables

**Table 1:** Demographic characteristics of sampled college students (*n* = 600).

Demographic Characteristics	*n*	%
Only child or not		
Yes	265	44.2
No	335	55.8
Gender		
Male	299	49.8
Female	299	49.8
Transgender	1	0.2
Unknown	1	0.2
Family origins		
City	242	40.3
Rural areas	358	59.7
Physical health status		
Good	352	58.6
Fair	232	38.7
Poor	16	2.7
Sleep quality		
Good	312	52.0
Fair	244	40.7
Poor	44	7.3
Academic performance		
Good	197	32.8
Fair	353	58.8
Poor	50	8.4
Father’s physical status		
Good	320	53.3
Fair	240	40.0
Poor	33	5.5
Unknown	7	1.2
Father’s mental health status		
Good	418	69.7
Fair	135	22.5
Poor	11	1.8
Unknown	36	6.0
Mother’s physical health status		
Good	280	46.7
Fair	268	44.7
Poor	50	8.3
Unknown	2	0.3
Mother’s mental health status		
Good	403	67.2
Fair	167	27.8
Poor	15	2.5
Unknown	15	2.5
Parents’ marital status		
Harmonious	335	55.8
Sometimes quarrel	225	37.5
Others (separated/divorced/one or both parents passed)	40	6.7

**Table 2:** Chi-square analysis of associated factors of depressive symptoms among college students.

Variables	Depressive Symptoms		
	*n* (%)	*χ* ^2^	*p*
Only child or not		0.92	0.337
Yes	88 (33.2)		
No	99 (29.6)		
Gender		2.48	0.629
Male	93 (31.0)		
Female	93 (31.0)		
Transgender	0 (0.0)		
Unknown	1 (100.0)		
Family origins		8.87	0.003
City	92 (38.0)		
Rural area	95 (26.5)		
Physical health status		48.83	<0.001
Good	72 (20.5)		
Fair	104 (44.8)		
Poor	11 (68.8)		
Sleep quality		33.51	<0.001
Good	70 (22.4)		
Fair	90 (36.9)		
Poor	27 (61.4)		
Academic performance		38.80	<0.001
Good	44 (22.3)		
Fair	109 (30.9)		
Poor	34 (68.0)		
Father’s physical health status		14.92	0.001
Good	83 (25.9)		
Fair	88 (36.7)		
Poor	16 (48.5)		
Unknown	0 (0.0)		
Father’s mental health status		18.40	<0.001
Good	110 (26.3)		
Fair	52 (38.5)		
Poor	7 (63.6)		
Unknown	18 (50.0)		
Mother’s physical health status		28.41	<0.001
Good	59 (21.1)		
Fair	108 (40.3)		
Poor	18 (36.0)		
Unknown	2 (100.0)		
Mother’s mental health status		27.56	<0.001
Good	98 (24.3)		
Fair	78 (46.7)		
Poor	5 (33.3)		
Unknown	6 (40.0)		
Parents’ marital status		12.80	0.002
Harmonious	85 (25.4)		
Sometimes quarrel	84 (37.3)		
Separated/divorced/one or both parents passed	18 (45.0)		

**Table 3:** Logistic regression analysis of the association between mental health knowledge level and depressive symptoms among college students.

Variables	Model 1				Model 2				Model 3			
	*B*	*Wald*	*OR* (95%CI)	*p*	*B*	*Wald*	*OR* (95%CI)	*p*	*B*	*Wald*	*OR* (95%CI)	*p*
Mental health knowledge level												
0~15	-	4.98	1.00	-	-	6.24	1.00	-	-	5.60	1.00	-
15~17	−0.40	2.15	0.67 (0.39~1.14)	0.142	−0.38	1.67	0.68 (0.38~1.22)	0.197	−0.34	1.18	0.72 (0.39~1.31)	0.278
17~18	−0.59	4.07	0.56 (0.31~0.98)	0.044	−0.65	4.12	0.52 (0.28~0.98)	0.042	−0.66	3.92	0.52 (0.27~0.99)	0.048
18~20	−0.51	3.89	0.60 (0.36~0.99)	0.048	−0.66	5.31	0.52 (0.30~0.91)	0.021	−0.63	4.30	0.53 (0.30~0.97)	0.038

Model 1: Unadjusted model with covariates; Model 2: Adjusted model with personal associated factors (family origins, physical health status, sleep quality, and academic performance); Model 3: Adjusted model with personal associated factors and family-associated factors (family origins, physical health status, sleep quality, and academic performance; parents’ physical health status, parents’ mental health status, and parents’ marital status).

## Data Availability

The data are not publicly available due to privacy and research ethical restrictions.
